# Immunogenicity and safety of influenza vaccination in patients with juvenile idiopathic arthritis on biological therapy using the microneutralization assay

**DOI:** 10.1186/s12969-017-0190-0

**Published:** 2017-08-07

**Authors:** M. S. Camacho-Lovillo, A. Bulnes-Ramos, W. Goycochea-Valdivia, L. Fernández-Silveira, E. Núñez-Cuadros, O. Neth, P. Pérez-Romero

**Affiliations:** 1 Unidad de Enfermedades Infecciosas e Inmunopatologías Pediátrica, Hospital Universitario Virgen del Rocío/Instituto de Biomedicina de Sevilla (IBIS), Sevilla, Spain; 2 Instituto de Biomedicina de Sevilla (IBIS), University Hospital Virgen del Rocío/CSIC/University of Sevilla, Unidad Clínica de Enfermedades Infecciosas, Microbiología y Medicina Preventiva, Sevilla, Spain; 3 Unidad de Reumatología Pediátrica, Hospital Materno Infantil, Hospital Regional Universitario de Málaga, Málaga, Spain

## Abstract

**Background:**

Seasonal influenza virus vaccination should be considered in all pediatric patients with rheumatic diseases. Few studies have addressed influenza vaccination safety and efficacy in this group. We aim to prospectively evaluate immunogenicity and safety of the trivalent inactivated influenza vaccine including A/H1N1, A/H3N2 and B strains in children with juvenile idiopathic arthritis (JIA) receiving biological therapy.

**Methods:**

Thirty-five children diagnosed with JIA and 6 healthy siblings were included. Serum samples were collected prior to, 4-8 weeks and one year after vaccination. Microneutralization assays were used to determine neutralizing antibody titers. The type and duration of therapy were analyzed to determine its effect on vaccine response. Clinical data of the participants were collected throughout the study including severe adverse events (SAE) and adverse events following immunization (AEFI).

**Results:**

Twenty-five patients (74.3%) received biological treatment for JIA; anti TNF-α was prescribed in 15, anti IL-1 receptor in 4 and anti IL-6 receptor therapy in 6 children. The seroprotection rate 4-8 weeks after vaccination in the JIA group was 96% for influenza A/(H1N1)pdm and influenza A/H3N2, and 88% for influenza B. No differences were found in GMT, seroprotection and seroconversion rates for the three influenza strains between the control group and patients receiving biological therapy. Furthermore, long-term seroprotection at 12 months after vaccination was similar in patients receiving either biological or non-biological treatments. No SAEs were observed.

**Conclusions:**

In this study, influenza vaccination was safe and immunogenic in children with JIA receiving biological therapy.

**Electronic supplementary material:**

The online version of this article (doi:10.1186/s12969-017-0190-0) contains supplementary material, which is available to authorized users.

## Background

Children and adolescents with rheumatic diseases (RD) are at increased risk of infection compared to age- and gender-matched subjects without RD due to their aberrant immunity and frequent use of immunosuppressive drugs [1]. Annual vaccination against influenza is recommended for immunocompromised patients [2]. Efficacy and safety of vaccination remain to be defined in rheumatologic patients with immunomodulatory therapy, including high doses of steroids. Recommendations for vaccination in pediatric patients with RD were published in 2011 by the European League Against Rheumatism (EULAR). Seasonal influenza virus vaccination should be considered in all pediatric patients with RD [3, 4]. Few studies have addressed influenza vaccination safety and efficacy in this group, most of which have small sample sizes, include a wide variety of rheumatic diseases and have scarce data on new biological therapies [3–6].

Juvenile idiopathic arthritis (JIA) is the most common inflammatory rheumatic chronic disease of childhood, with a prevalence of approximately 1 per 1000. JIA is defined as arthritis of unknown etiology beginning prior the age of 16 years persisting for at least 6 weeks with other known conditions excluded [7]. The incidence of influenza infection in this patient group is unknown and evidence is lacking whether children with JIA have an increased risk of severe influenza infection or its associated complications. However, influenza virus can trigger disease flare, treatment interruption and the use of antibiotic therapy for suspected bacterial co-infection, all of which are associated with unnecessary medical revisions, thus reducing the quality of life of these children and their families [8]. The development of therapeutic biological agents including anti IL-6 and anti IL-1 receptor (R) therapy, has markedly altered treatment strategies for RD and greatly improved the prognosis of affected individuals [9]. Whilst influenza vaccination in children with RD receiving non biological immunosuppressive agents results in similar serum antibody titers compared to those of healthy children, few data exist on the impact of biological therapy [10]. Here, we describe the immunogenicity and safety of influenza vaccination and long term seroprotection on a pediatric cohort diagnosed with JIA receiving immunomodulatory therapy including anti IL-6 and anti IL-1R therapy and review the published literature.

## Methods

### Patient inclusion

We performed a prospective, longitudinal study of children with JIA receiving the influenza vaccine in two consecutive influenza seasons 2013/2014 and 2014/2015 at the University Hospital Virgen del Rocío, Seville. Diagnosis of JIA was made according to the recommendations of the International League of Associations for Rheumatology [7]. Patients (aged 1-18 years) and healthy siblings, as controls, were included once and consecutively after the legal guardians signed informed consent. Exclusion criteria for this study included acute infection at the time of vaccination, history of previous adverse reaction or anaphylaxis to chicken egg protein or any other vaccine, demyelinating disease or parents refusing to sign the informed consent. The Ethics Committee for Clinical Research of the local Hospital approved the study.

### Samples

Serum samples were collected from each patient at the time of vaccination (baseline), at 4-8 weeks post vaccination (Tpv) and one year after vaccination (before the new seasonal vaccine immunization). Samples were stored at −80 °C for further analysis.

### Clinical and laboratory data

Demographic data, including sex, age, time of diagnosis, JIA subtype, type of treatment, previous influenza vaccination, date of vaccination, activity of disease using the Juvenile Arthritis Disease Activity Score (JADAS) including 4 criteria (physician global assessment of disease activity, parent/patient global assessment of well-being, active joint count, and erythrocyte sedimentation rate), full blood count, immunoglobulin levels, Rheumatoid factor (RF), antinuclear antibodies (ANA), immunological and clinical response were collected [11]. Patients diagnosed with JIA were stratified according to the treatment received at time of vaccination (biological treatment and non-biological treatment including methotrexate (MTX) only or no treatment).

### Safety assessment

Patients were followed-up during a six-month period to collect and record adverse events following immunization (AEFI). International guidelines for AEFI reporting and causality assessment were used according to the WHO, Global Vaccine Safety Initiative (GVSI) [12]. AEFI were monitored using a questionnaire given to patients on the vaccination day, and every 3 months in the follow-up visits, whilst serious adverse events (SAEs) and adverse events of special interest (AESI) were actively monitored during trial duration. AESI included were flare and worsening of the JADAS [11]. A flare was defined as a worsening of 40% in at least 2 of the 6 disease activity parameters of the American College of Rheumatology (ACR) pediatric core set, without a simultaneous improvement of 30% or more in at least 2 of the remaining parameters [13].

### Vaccines

Patients received one dose of the trivalent non-adjuvanted inactivated vaccine in 2013/2014 (Sanofi, Sanofi- Pasteur MSD) containing the following strains: influenza A/California/7/2009-H1N1 (A/(H1N1)pdm), influenza A/Victoria/361/2011-H3N2 (A/H3N2) and B/Massachusetts/2/2012 and in 2014/2015 containing the following strains: A/California/7/2009 (H1N1)pdm; A/Texas/50/2012 (H3N2); B/Massachusetts/2/2012. Children under the age of nine, who were vaccinated for the first time against influenza, received a second dose of vaccine after one month.

### Microneutralization assay

As described previously [14], two-fold serial dilutions of the inactivated human sera (from 1:5 to 1:2560) were incubated for 2 h at 37 °C with a multiplicity of infection (MOI) of 0.25 for A/(H1N1)pdm, 1.4 MOI for A/H3N2, and 0.05 MOI for B. Four wells with infected cells were used as positive controls and wells with only cells were used as negative controls. One hundred microliters containing 1.5 × 10^5^ Madin–Darby canine kidney (MDCK) cells/ml were added to each well of a 96-well dish and incubated for 24 h at 37 °C. Primary antibodies for influenza A nucleoprotein (Anti-Influenza A nucleoprotein Bioporto Bionova, Gentofte, Denmark) diluted 1:1500 or influenza B nucleoprotein (Anti-Influenza B nucleoprotein Bioporto from Bionova, Gentofte, Denmark) diluted 1/1000 were used, followed by a HRP-conjugated anti-mouse IgG antibody diluted 1:1000 (Sigma-Aldrich). One-hundred microliters of peroxidase substrate (3, 3′, 5, 5′-Tetramethyl-benzidine substrate, supersensitive, for ELISA; Sigma-Aldrich) were added to each well and absorbance was measured at 450 nm. The average absorbance (*A*450) from the quadruplicate wells of virus-infected (VC) and uninfected (CC) control wells was determined, and the neutralizing endpoint was determined by using a 50% specific signal calculation. The endpoint titer was expressed as the reciprocal of the highest dilution of serum with *A*450 value less than *X*, where *X* = [(average *A*450 of VC wells) - (average *A*450 of CC wells)]/2 [14]. Sera were considered positive if titers were ≥40 obtained in at least two independent assays. Vaccination immunogenicity parameters were based on the following international EMEA/CPMP 1997 criteria described for the hemagglutination inhibition assay, which have shown correlation with antibody titres measured by microneutralization assay [15, 16]. Seroprotection was considered as a post-vaccination serum antibody titer >1:40. Seroconversion was considered when a 4-fold antibody titer increase from baseline was achieved. Geometric mean titers (GMT) defined as mean antibody titer in the group of vaccinated individuals; seroprotection rate defined as the proportion of vaccinated individuals with antibody titers ≥1:40; seroconversion rate as the percentage of subjects showing seroconversion; and geometric mean ratio (GMR) defined as seroconversion factor post to prevaccination.

### Statistical analysis

A descriptive statistical analysis was performed. Continuous variables were expressed as median and interquartile range or mean ± standard deviation if adjusted to normal distribution, and evaluated by Shapiro-Wilk or Kolmogorov-Smirnov tests when appropriate. The main primary outcome for the analysis was seroprotection. Secondary outcomes were seroconversion, GMT after vaccination, and safety. For bivariate analysis, the chi-square test, Fisher’s exact test or the McNemar test were used for categorical variables. For quantitative variables, the Mann-Whitney test or Student’s t test were used. If the variance was not homogeneous the Welch test was applied, in case of homogeneity of variances, ANOVA was applied.

For immunogenicity analysis, GMT at each time point was used. Relative risk and 95% confidence interval (CI) were calculated by taking the exponent of natural logarithm of the mean and 95% CI. Results were analyzed by PASW Statistic 18.0.1 software. Statistical significance was established as a *p* value of <0.05. All reported *p* values were based on two-tailed tests. Bivariate analysis was an exploratory outcome and adjustment for multiplicity was done for this analysis using the Bonferroni method (adjusted *p* value <0.002).

## Results

### Patient cohort

Baseline clinical parameters and demographic characteristics of the patients are shown in Table 1. A total of 41 subjects were included in the study. Three patients received two vaccine doses. 35 (83.3%) patients were diagnosed with JIA (experimental group), median age of 10.6 years (IQR 8.8-12.7), 12 (35.3%) were male and 25 (74.3%) were receiving biological treatment for JIA; 15 patients anti-TNFα (11 etanercept and 4 adalimumab), 4 anti-IL-1R (anakinra) and 6 anti-IL-6R (tocilizumab). Six (14.2%) children were healthy individuals (control group), with a median age of 11.6 years (IQR 9.8-14.6) and two (33.3%) male.

**Table 1:** Baseline clinical and laboratory parameters

Variables	Control group (*n* = 6)	JIA group (*n* = 35)	Biological therapy (*n* = 25)	Non biological therapy (*n* = 10)
Female n (%)	4 (66.6)	23 (65.7)	17 (68)	6 (60)
Age in y (IQR)	11.6 (9.8-14.6)	10.6 (8.8-12.7)	11.4 (8.9-13.0)	11.4 (4.7-12.3)
Previous seasonal vaccination n (%)	2 (33)	22 (62.8)	18 (72)	4 (40)
Duration of JIA in y (IQR)		4.9 (2.7-8.3)	5.99 (3.0-8.6)	3.94 (2.5-4.7)
JIA category n (%)				
Persistent oligoarthritis		13 (37.1)	7 (28)	6 (60)
Extended oligoarthritis		6 (17.1)	5 (20)	1 (10)
Polyarthritis RF negative		6 (17.1)	4 (16)	2 (20)
Polyarthritis RF positive		0 (0)	0 (0)	0 (0)
Systemic onset arthritis		7 (20)	7 (28)	0 (0)
Enthesitis related arthritis		3 (8.5)	2 (8)	1 (10)
Psoriatic arthritis		0	0	0
Undifferentiated arthritis		0	0	0
Treatment n (%) ^(a)^				
Without therapy		3 (8.5)	_	3 (30)
Systemic corticosteroids (concomitant MTX + tocilizumab)		2 (5.7)	2 (8)	
Methotrexate monotherapy		7 (20)	_	7 (70)
Biological monotherapy		16 (45.7)	16 (64)	_
Biological therapy + methotrexate		9 (25.7)	9 (36)	_
Duration of biological therapy in years (IQR)	NA	NA	3.1 (1.8-4.8)	NA
JADAS 71 (IQR)		1 (0-5)	3 (0-7)	2 (0.25-6.75)
IgG mg/dl (IQR)		1036 (888-1243)	1021 (880-1249)	1143 (965-1205)
Leukocytes ×10^9^/L (IQR)	6.5 (3.7-8.2)	6.0 (5.2-7.4)	5.8 (5.0-6.9)	7.5 (5.9-9.1)
Lymphocytes ×10^9^/L	2.3 (1.3-2.5)	2.2 (1.6-2.8)	2.2 (1.6-2.8)	2.283 (1.9-3.1)
Neutrophils ×10^9^/L	3.4 (2.0-4.7)	3.1 (2.7-3.9)	3.1 (2.5-3.6)	3.9 (3.0-4.7)
Hemoglobin g/L (IQR)	13.3 (12.8-13.6)	13.3 (12.7-13.6)	13.3 (12.8-14.2)	13.1 (12.2-13.3)
Platelets ×10^9^/L (IQR)	305 (289-376)	263 (234-320)	255 (227-318)	275 (251-356)
ESR mm/h (IQR)	5 (4.0-9.5)	8 (4.0-10.5)	6 (4.0-9.0)	8.5 (6.5-10.8)

Values are expressed as median unless stated otherwise; *IQR* interquartile range, *JIA* juvenile idiopatic arthritis; *y* years; *MTX* methotrexate, *IgG* immunoglobulin G, *ESR* erythrocyte sedimentation rate, *NA* not applicable

^(a)^Methotrexate was given at a dose of 10-15 mg/m^2^/week (35% received lower doses due to clinical remission). Systemic corticosteroids included oral 0.1 mg/kg/day in a patient and 10 mg/kg/dose iv in the remaining patients. Biological therapy included: tocilizumab 8 mg/kg/dose monthly or fortnightly (polyarticular or systemic JIA respectively), anakinra 2-4 mg/kg/day, etanercept 0.8 mg/kg/week and adalimumab 24 mg/kg/m^2^ (52% of patients received lower doses due to clinical remission). Biological therapy was administered simultaneously with the influenza vaccination

### Immunological response to influenza vaccination

Overall, control and experimental, groups together achieved an adequate seroprotection rate at Tpv: 97.8% for influenza A/(H1N1)pdm, 95.6% for influenza A/H3N2 and 91.1% for influenza B. Median Tpv was 5.4 weeks IQR (4.6-6.1). Immunological response to vaccination was analyzed in the experimental group and compared with the control group. No differences were found in GMT, seroprotection and seroconversion rates at Tpv for the three influenza strains (A/(H1N1)pdm, A/H3N2 and B) (Table 2). Children diagnosed with JIA were stratified according to receiving biological treatment and again no differences were observed in the antibody response to vaccination based on treatment (Table 2). There were no differences for A/(H1N1)pdm and A/H3N2 postvaccination seroprotection rates between children receiving biological treatment and those receiving no biological therapy (96% vs. 100%; *p* = 0.521); neither for influenza B strain (88% vs. 90%; *p* = 0.867). Similarly, receiving biological treatment was not associated with differences in the post-vaccination GMT or in seroconversion for the three vaccine strains. Two of the patients receiving biological treatment had concomitant systemic steroids, however no differences were found in pre- or post-vaccination seroprotection or antibody titers compared with patients on biological treatment with no steroids.

**Table 2:** Influenza A/(H1N1)pdm, A/H3N2 and B virus antibody response in JIA patients receiving or not biological therapy

	Control group (*n* = 6)	JIA group (*n* = 35)	*p*-value	Biological therapy (*n* = 25)	No biological therapy (*n* = 10)	*p-*value
A/H1N1						
GMT						
Pre-vaccine (range)	31.7 (10 -160)	47.8 (5-1280)	*0.388*	45.9 (5 -320)	52.7 (5 -1280)	*0.504*
Post-vaccine (range)	320 (160-1280)	273.1 (20-2560)	*0.684*	242.5 (20-2560)	367.5 (40-2560)	*0.535*
GMR mean (range)	6.10 (2.7-22.3)	5.1 (2.7-85.2)	*0.646*	4.89 (2.71-85.2)	5.99 (2.71-30.2)	*0.510*
Seroprotection						
Pre-vaccine- n (%)	3 (50)	24 (68.6)	*0.375*	18 (72.0)	6 (60)	*0.490*
Post-vaccine- n (%)	6 (100)	34 (97.1)	*0.675*	24 (96)	10 (100)	*0.521*
Seroconversion- n(%)	5 (83.3)	23 (65.7)	*0.391*	15 (60)	8 (80)	*0.260*
A/H3N2						
GMT						
Pre-vaccine (range)	22.44 (5-80)	38.44 (5-2560)	*0.562*	34.82 (5-80)	49.2 (5-2560)	*0.334*
Post-vaccine (range)	320 (80-2560)	233.0 (20-2560)	*0.684*	199.7 (20-2560)	342.9 (80-2560)	*0.565*
GMR mean (range)	8.0 (3.1-35.0)	4.9 (2.7-22.3)	*0.079*	4.70 (2.7-13.7)	5.66 (2.7-22.4)	*0.383*
Seroprotection						
Pre-vaccine- n (%)	3 (50)	22 (62.9)	*0.551*	17 (68.0)	5 (50.0)	*0.319*
Post-vaccine- n(%)	6 (100)	34 (97.1)	*0.675*	24 (96)	10 (100)	*0.521*
Seroconversion- n(%)	5 (83.3)	22 (62.9)	*0.328*	15 (60)	7 (70)	*0.580*
Flu B						
GMT						
Pre-vaccine (range)	63.4 (40-80)	40.8 (5-640)	*0.114*	26.1 (5-640)	37.3 (5-320)	*0.912*
Post-vaccine (range)	640 (40-2560)	118.9 (20-1280)	*0.396*	91.9 (20-1280)	226.2 (20-1280)	*0.871*
GMR mean (range)	4.7 (2.7-8.3)	3.8 (2.7-9.8)	*0.235*	3.6 (2.7-9.9)	4.7 (3.06-6.70)	*0.038*
Seroprotection						
Pre-vaccine n (%)	6 (100)	24 (68.6)	*0.108*	17 (68.0)	7 (70)	*0.908*
Post-vaccine n (%)	6 (100)	31 (88.6)	*0.383*	22 (88.0)	9 (90.0)	*0.867*
Seroconversion n (%)	4 (66.7)	17 (48.6)	*0.413*	9 (36)	8 (80)	*0.019*

Parameters were compared by multiple comparison chi-square test or linear regression. *GMR* geometric mean ratio, *GMT* geometric mean titer

Within the experimental group, patients were stratified according to the type of biological treatment and no differences were found in the antibody response against the three influenza strains (Additional file 1: Table S1).

Three children received two vaccine doses and showed, post-vaccination seroprotection after the second dose, however antibody titers did not differ from those receiving one dose only (data not shown).

### Long-term seroprotection rates after vaccination

In the JIA group 12 individuals had serum samples available one year after vaccination, six (50%) of them received biological treatment, and long-term seroprotection after vaccination was analyzed. Overall no differences in humoral responses were observed for the three strains analyzed. All 12 patients (100%) were seroprotected at Tpv vs. 91.7% one year after vaccination against A(H1N1)pdm (*p* = 0.327); 81.8% vs. 91.7% were protected against A/H3N2 (*p* = 0.070), and 91.7% vs. 91.7% (*p* = 0.500) against influenza B at Tpv vs. one year after vaccination.

Similarly, the type of treatment (biological or non-biological) did not influence long-term antibody response. In patients with biological treatment (*n* = 6), seroprotection at one-year post vaccination was 100% (100% at Tpv) for influenza A/H1N1 (*p* = 0.999), 66.7% (83.3% at Tpv) for H3N2 (*p* = 0.999) and 100% (100% at Tpv) for influenza B (*p* = 0.999).

### Bivariate analysis

The analysis of confounding factors associated with antibody response to vaccination in patients with JIA (Additional file 2: Table S2), revealed seroprotected patients to Tpv A/H1N1 to have lower platelet levels and ESR at baseline compared with non-seroprotected individuals (286 vs. 594 × 10 [9]/L, *p* = 0.001 and 7.2 vs 18 mm/h, *p* = 0.009). When adjusted for multiplicity (*p* < 0.002), only platelet level was significantly lower in seroprotected patients to Tpv for A/H1N1.

### Safety of influenza vaccination

There was no follow-up loss of any of the subjects in the study. Overall no SAEs were observed. Seven out of 41 (17%) children showed adverse drug local reactions (six with local skin inflammation and one hematoma), whilst two out of 41 (4.9%; one each in the control and biological therapy group) had systemic adverse drug reactions (general malaise and fever >24 h). During the 6-month follow-up, 16 febrile episodes, not related to vaccination, were reported in 11 patients (four in the non-biological and seven in the biological therapy group), 10 of which were treated with oral antibiotic therapy. JADAS score increased in 6 patients at Tpv compared with baseline, in three of them from a low or no activity to moderate [17]. However, none of them had a flare based on the mentioned criteria [13].

## Discussion

In this cohort of children with JIA receiving immunomodulatory therapy, influenza vaccination was safe and immunogenic; and the neutralizing antibody response was independent of the type of treatment. To date few influenza vaccine studies have included children with JIA, and biological therapy was specifically studied in only five of them (Table 3). In all but one (Toplak et al. [18]) the hemagglutination inhibition test (HAI) was used to determine antibody response to vaccination, which is known for its overall poor sensitivity, thereby potentially underestimating the seroprevalence in a given population [19]. The microneutralization assay, which was used in the present study, has been shown to enhance sensitivity and specificity [14]. No correlate of protection has been defined for microneutralization, but good correlation with HAI defined protection thresholds has been reported [16]. Furthermore, it has been proposed that HAI antibody seroprotection cut-offs in children aged 6-72 months may be 1:110 to achieve 50% protection [20], thus it is possible that children with RD may need antibody titers equal to or higher than those described. There is a need to review the current practice of evaluating vaccine responses and should include more advanced techniques to evaluate immunogenicity such as microneutralization assay, which identifies functional antibodies, rather than quantitative tests only [21].

**Table 3:** Summary of published reports describing pediatric patients with JIA receiving influenza vaccine

Article	Vaccination season Vaccine type	Methodology antibody response	Number of JIA patients	Number of controls	Number of JIA patients on biological therapy	Adverse events (AE)	Efficacy (seroprotection) according to group and vaccine type	Time post-vaccination
Toplak et al. [18]*.* 2012 Slovenia	2008/2009 A/(H1N1)pdm, H3N2,B	Virus neutralization test	31	14	anti-TNFα 4	Short-term AE: JIA 35% Control 36%	JIA 100%H1N1 100% H3N2 84%B	1 month
						Pain at injection site: JIA 32% Control 21%	anti-TNFα 75%H1N1 75%H3N2 10% B	
						Malaise and headache: JIA 3% Control 14% No severe AE	Control 100% H1N1 100%H3N2 78%B	
Malleson et al. [6]. 1993 Canada	1991/1992-2008/2009 A/(H1N1)pdm, H3N2,B	HAI	26	13	0	Soreness and redness at injection site: JIA 69% and 15% Control 69% and 8%	JIA 100% H1N1 97% H3N2 97% B	4 - 6 week
						Fever and feeling unwell: JIA 19% and 46% Control 8% and 26% No severe AE	Control 90% H1N1 90% H3N2 90% B	
Dell’ Era et al. [25]. 2012 Italy	2010/2011-2008/2009 A/(H1N1)pdm, H3N2,B	HAI	60	30	Etanercept 30	Local AE: JIA with DMARD 43.3% JIA with Etanercept 36.7% Control 40% Systemic AE: JIA with DMARD 30% JIA with Etanercept 26.7% Control 26.7% Severe AE: JIA with DMARD 3.3% JIA with Etanercept 3.3%	JIA with DMARD 100% H1N1 100% H3N2 83.3% B JIA with Etanercept 100% H1N1 100% H3N2 30% B Control 100% H1N1 100% H3N2 93,3% B	1 month
Aikawa et al. [26]. 2013 Brazil	2009 A/(H1N1)pdm	HAI	95	91	anti-TNFα 16	Local pain: JIA 21.1% Control 23.1%	JIA 88.4% H1N1 anti-TNFα 100% H1N1 Control 95.6% H1N1	3 weeks
						No severe AE		
Shinoki et al. [9]. 2012 Japan	2007/2008 A/(H1N1)pdm, H3N2,B	HAI	27	17	Tocilizumab 27	Local AE: JIA14.8% Control 0%	JIA 88.9% H1N1 85.2% H3N2 40.7% B	4 -7 weeks
						No severe AE	Control 76.5% H1N1 100% H3N2 35.3% B	
Miraglia et al. [5]. 2011 Brazil	2009 A/(H1N1)pdm	HAI	83	-	Not specified	1.3% redness, 20% malaise JIA	JIA 85.5% H1N1	3 weeks
						No severe AE		
Carvalho et al. [8]. 2013 Brazil	2008 A/(H1N1)pdm, H3N2,B	HAI	44	10	anti-TNFα 5	Local pain JIA 13.6% Control 10% Other local change JIA4.5% Control 10%	JIA 100% H1N1 93% H3N2 95% B Anti TNFα 60% H1N1 100% H3N2 80% B Control 100% H1N1 80% H3N2 100 B	30-40 days

*HAI* hemagglutination inhibition, *DMARDS* disease-modifying anti-rheumatic drugs. Severe AE: defined as patients requiring hospitalization or iv treatment

Blockade of the IL-6R has been shown to decrease lymphocyte activation and to restore B and T cell homoeostasis in patients with SLE [22]. Anti-IL-6R treatment may diminish influenza vaccine antibody responses since IL-6 induces B cell differentiation into antibody-forming cells and T cells into effectors cells [22]. However, Shinoki et al. studied JIA patients treated with tocilizumab (anti IL-6R) and demonstrated that influenza vaccination was immunogenic and safe, as was the case in our study (Table 3) [9]. Whilst in healthy subjects therapy with anti-IL-1 did not affect the antibody responses after vaccination with influenza vaccine [23], the impact of its use in children with JIA is unknown. To the best of our knowledge, this is the first study evaluating influenza vaccine in children receiving IL-1R antagonist (anakinra) for JIA and was found to be safe and immunogenic. However, further studies are needed to confirm its safety in pediatric patients, as only four patients were included here.

Anti-TNF-α therapy in rheumatoid arthritis inhibits germinal center reactions, thereby leading to decrease peripheral blood memory B cell levels, potentially contributing to a poor response to vaccination [24]. Four pediatric studies (Table 3) have evaluated influenza vaccine in JIA patients receiving anti-TNF-α therapy. Antibody titers against A(H1N1), A(H3N2), and B vaccine viruses were found to be reduced in patient receiving anti-TNF-α although only four children were enrolled [18]. In another study, 30 JIA patients treated with anti-TNF-α showed significantly lower GMTs against the A/H1N1 strain compared to those treated with disease-modifying antirheumatic drugs (DMARD) and healthy controls. Furthermore, sero-conversion and -protection rates were significantly lower in patients receiving anti-TNF-α [25], as was also reported by Carvalho et al. in 5 patients and only for the A/H1N1 strain [8]. In the Aikawa et al. study, no differences were observed in immunogenicity parameters between patients with or without anti-TNF-α blockers however, the seroconversion rate for the A/H1N1 strain was significantly lower in JIA patients compared to controls (83.2% vs. 95.6%, *p* = 0.008) [26]_._ In our study, no differences between the control group and patients with or without biological therapy were observed, thus biological therapy plus MTX did not alter the vaccine response. Although lack of differences between groups could be due to the small sample size, these findings are in contrast with previous results, where the use of anti-TNF-α blockers was associated with a reduced vaccine response [25, 26]. One year after vaccination seroprotection rates were similar with a slight and expectable reduction for all strains but A/H3N2, which was higher. This could be due to natural exposition to the virus, or test precision variability. Although it was evaluated in only few patients, biological treatment seems not to influence in long-term seroprotection rates after vaccination.

Like in other studies in children with RD, no SAEs were reported and vaccination using an inactivated influenza vaccine was shown to be safe with mild systemic and local reactions as most reported AEFI [26, 27]. Our patients remained clinically stable (JADAS-71:1 (0-5)) during the study period with only two patients receiving systemic corticoids at the time of vaccination. Lack of active inflammation at the time of vaccination could have influenced the safety and immunogenicity outcomes in our cohort. This is supported by the study of Toplak et al., where only 42% of the children had inactive disease at the time of vaccination and all children suffering from a flare post vaccination, had at least one criterion for active disease prior to vaccination [18]. This is important as high dose steroid therapy is known to be associated with a reduced immnunogenicity of the influenza vaccine resulting in lower antibody titers [28, 29]. We observed seroprotected patients to have lower inflammatory markers such as platelet count and ESR compared to non-seroprotected children. A low inflammatory state may result in an enhanced cellular immune response (Th1 vs Th2) thereby supporting B and T cell differentiation with subsequent improved vaccine response [30, 31]. In contrast to the studies of Dell’ Era and Toplak et al. where only 8.3% and 13% of the patients were previously vaccinated, 62.8% of children with JIA (and 72% of those receiving biological treatment) received the influenza vaccine the season prior to this study, and that has likely contributed to the overall high vaccine response shown here. This is also supported by previous results suggesting that baseline seroprotection promotes significantly higher GMT after vaccination [32]. Whilst over 60% (H1N1 68.6%, H3N2 62.9% and Flu B 68.6%) of our JIA children were seroprotected prior vaccination, only 20% of the patients described by Toplak and Aikawa et al. showed pre-vaccine seroprotection, further supporting the benefit of seasonal influenza vaccination. However, differences between participant’s age and previous exposure to the virus could alter pre-vaccination seroprotection rates. A limitation of this study is the overall low number of patients and healthy controls included, which may have limited the power to detect differences, especially when performing subgroup analysis. Although clinical, laboratory baseline parameters and GMT baseline titers were similar in the groups; there was heterogeneity in the treatment used and likely in the previous influenza exposure (artificial or natural) of the included patients. Further studies evaluating vaccine efficacy and effectiveness are necessary.

## Conclusions

In this prospective study of children with JIA, the use of the trivalent inactivated influenza vaccine including A/H1N1, A/H3N2 and B strains was shown to be safe and immunogenic. The type of immunomodulatory therapy did not alter vaccine response. This patient group would likely benefit from seasonal influenza vaccination thereby supporting the annual immunization program.

## Additional files

Additional file 1: Table S1. Influenza A/(H1N1)pdm, A/H3N2 and B virus antibody response in JIA patients according to type of biological therapy. (DOCX 42 kb)

Additional file 2: Table S2. Multivariable analysis for Post-vaccine seroprotection in patients with Juvenile Idiopathic Arthritis. (DOCX 18 kb)

## Abbreviations

AEFI: adverse events following immunization
AESI: Adverse events of special interest
EULAR: European League Against Rheumatism
GMR: Geometric mean ratio
GMT: Geometric mean titers
GVSI: Global Vaccine Safety Initiative
JADAS: Juvenile Arthritis Disease Activity Score
JIA: juvenile idiopathic arthritis
SAE: severe adverse events
Tpv: time post vaccination
