# Supplementary material for: Immunogenicity and safety of influenza vaccination in patients with juvenile idiopathic arthritis on biological therapy using the microneutralization assay
Source: Pediatr Rheumatol Online J. 2017 Aug 7;15:62. doi: 10.1186/s12969-017-0190-0 (PMC5547451; doi:10.1186/s12969-017-0190-0)
Supplement: Supplementary file 1 — Influenza A/(H1N1)pdm, A/H3N2 and B virus antibody response in JIA patients according to type of biological therapy. (DOCX 42 kb) [file 12969_2017_190_MOESM1_ESM.docx]

**Additional file 1: Table S1. Influenza A/(H1N1)pdm, A/H3N2 and B virus antibody response in JIA patients according to type of biological therapy**

|  | anti TNF-α (Etanercept + Adalimunab) | | | |
| --- | --- | --- | --- | --- |
|  |  |  |  |  |
| A(H1N1)pdm | **Yes (n=15)** | **No (n=10)** | ***p-*value** | **RR (95% CI)/ B coeff (95% CI)** |
| GMT |  |  |  |  |
| Pre-vaccine (range) | 66.49 (10-320) | 26.39 (5-80) | *0.026* | 0.21 (0.02-0.40) |
| Post-vaccine (range) | 278.57 (40-2560) | 196.98 (20-2560) | *0.519* | 0.05 (-0.11-0.22) |
| GMR | 4.07 (2.71-13.73) | 6.44 (2.71-85.23) | *0.163* | -0.18 ( -0.43-0.07) |
| Seroprotection- n(%) |  |  |  |  |
| Pre-vaccine - n(%) | 12 (80.0) | 6 (60.0) | *0.275* | 2.66 (0.44-15.95) |
| Post-vaccine - n(%) | 15 (100) | 0 (0) | *0.211* | 9 (0.73-1.10) |
| Seroconversion- n(%) | 9 (60.0) | 6 (60.0) | *0.999* | 1 (0.19-5.12) |
|  |  |  |  |  |
| A/H3N2 |  |  |  |  |
| GMT |  |  |  |  |
| Pre-vaccine (range) | 36.48 (5-80) | 32.49 (10-80) | *0.692* | 0.06 (-0.25-0.37) |
| Post-vaccine (range) | 201.58 (20-2560) | 196.98 (40-2560) | *0.967* | 0.003 (-0.16-0.16) |
| GMR | 4.62 (2.71-13.73) | 4.82 (2.71-13.73) | *0.845* | -0.04 (-0.47-0.39) |
| Seroprotection- n(%) |  |  |  |  |
| Pre-vaccine - n(%) | 10 (66.7) | 7 (70.0) | *0.861* | 0.85 (0.15-4.89) |
| Post-vaccine - n(%) | 14 (93.3) | 10 (100) | *0.405* | 1.07 (0.93-1.22) |
| Seroconversion- n(%) | 9 (60.0 ) | 9 (60.0) | *0.999* | 1.00 (0.19-5.12) |
|  |  |  |  |  |
| Flu B |  |  |  |  |
| GMT |  |  |  |  |
| Pre-vaccine (range) | 45,94 (20-80) | 37.32 (5-640) | *0.671* | 0.03 (-0.14 - 0.22) |
| Post-vaccine (range) | 76.38 (20-640) | 121.25 (20-1280) | *0.335* | -0,8 (-0.27-0.096) |
| GMR | 3.13 (2.71-4.36) | 4.43 (2.71-9.89) | *0.017* | -0.64 (-1.16- -0.12) |
| Seroprotection- n(%) |  |  |  |  |
| Pre-vaccine - n(%) | 11 (73.3) | 6 (60.0) | *0.484* | 1.83 (0.33-10.09) |
| Post-vaccine - n(%) | 14 (93.3) | 8 (80.0) | *0.315* | 3.50 (0.27-44.95) |
| Seroconversion- n(%) | 3 (20.0) | 6 (60.0) | *0.041* | 0.16 (0.02-0.99) |

|  | anti IL-1 Receptor (Anakinra) | | | |
| --- | --- | --- | --- | --- |
|  |  |  |  |  |
| A(H1N1)pdm | **Yes (n= 4)** | **No (n=21)** | ***p*-value** | **RR (95% CI)/B coeff (95% CI)** |
| GMT |  |  |  |  |
| Pre-vaccine (range) | 20 (5-40) | 53.83 (5-320) | *0.080* | -0.12 (-0.27-0.017) |
| Post-vaccine (range) | 95.13 (20-640) | 289.83 (40-2561) | *0.113* | -0.95 (-0.21-0,02) |
| GMR | 5.61 (2.71-23.41) | 4.76 (2.71-85.23) | *0.713* | 0,36 (-0.16-0.23) |
| Seroprotection- n(%) |  |  |  |  |
| Pre-vaccine - n(%) | 2 (50.0) | 16 (76.2) | *0.285* | 0.31 (0.03-2.82) |
| Post-vaccine - n(%) | 3 (75.0) | 21 (100) | *0.019* | 1.33 (0.75-2.34) |
| Seroconversion- n(%) | 2 (50.0) | 13 (61.9) | *0.656* | 0.61 (0.07-5.27) |
|  |  |  |  |  |
| A/H3N2 |  |  |  |  |
| GMT |  |  |  |  |
| Pre-vaccine (range) | 40 (40-40) | 33.91 (5-80) | *0.672* | 0.04 (-0.18 - 0.28) |
| Post-vaccine (range) | 160 (40-640) | 208.35 (20-2560) | *0.723* | -0.21 (-0.14 - 0.10) |
| GMR | 3.95 (2.71-5.76) | 4.86 (2.71-13.73) | *0.457* | -0.11 (-0.44 - 0.20) |
| Seroprotection- n(%) |  |  |  |  |
| Pre-vaccine - n(%) | 4 (100) | 13 (61.9) | *0.134* | 0.61 (0.44-0.86) |
| Post-vaccine - n(%) | 4 (100) | 20 (95.5) | *0.656* | 0.95 (0.86-1.04) |
| Seroconversion- n(%) | 2 (50.0) | 13 (61.9) | *0.656* | 0.61 (0.07-5.27) |
|  |  |  |  |  |
| Flu B |  |  |  |  |
| GMT |  |  |  |  |
| Pre-vaccine (range) | 40 (5-640) | 42.72 (5-320) | *0.920* | -0.007 (-0.14-0.13) |
| Post-vaccine (range) | 160 (40-1280) | 82.68 (20-640) | *0.302* | 0.07 (-0.06-0.20) |
| GMR | 4.86 (2.71-9.89) | 3.39 (2.71-6.43) | *0.073* | 0.37 (-0.03-0.78) |
| Seroprotection- n(%) |  |  |  |  |
| Pre-vaccine - n(%) | 2 (50.0) | 15 (71.4) | *0.400* | 0.40 (0.04-3.52) |
| Post-vaccine - n(%) | 4 (100 ) | 18 (85.7) | *0.420* | 0.85 (0.7-1.01) |
| Seroconversion- n(%) | 2 (50.0) | 7 (33.3) | *0.524* | 2.0 (0.23-17.33) |

|  | anti IL-6 Receptor (Tocilizumab) | | | |
| --- | --- | --- | --- | --- |
|  |  |  |  |  |
| A(H1N1)pdm | **Yes (n =6)** | **No (n= 19)** | ***p*-value** | **RR (95% CI)B coeff (95 CI)** |
| GMT |  |  |  |  |
| Pre-vaccine (range) | 31.74 (5-80) | 51.63 (5-320) | *0.328* | -0.08 (-0.26-0.09) |
| Post-vaccine (range) | 320 (40-2560) | 222.18 (20-2560) | *0.554* | 0.04 (-0.10-0.18) |
| GMR | 7.05 (2.71-85.23) | 4.35 (2.71-23.41) | *0.203* | 0.14 (-0.08-0.37) |
| Seroprotection- n(%) |  |  |  |  |
| Pre-vaccine - n(%) | 4 (66.7) | 14 (73.7) | *0.739* | 0.76 (0.10-5.60) |
| Post-vaccine - n(%) | 6 (100) | 18 (94.7) | *0.566* | 0.94 (0.84-1.05) |
| Seroconversion- n(%) | 4 (66.7) | 11 (57.9) | *0.702* | 1.45 (0.21-9.98) |
|  |  |  |  |  |
| A/H3N2 |  |  |  |  |
| GMT |  |  |  |  |
| Pre-vaccine (range) | 28.28 (10-80) | 37.18 (5-80) | *0.411* | -0.10 (-0.37- 0.15) |
| Post-vaccine (range) | 226.27 (80-2560) | 192.01 (20-2560) | *0.797* | 0.01 (-012-0.16) |
| GMR | 5.49 (2.71-13.73) | 4.78 (2.71-13.73) | *0.386* | 0.16 (-0.21-0.53) |
| Seroprotection- n(%) |  |  |  |  |
| Pre-vaccine - n(%) | 3 (50) | 14 (73.7) | *0.278* | 0.28 (0.04-2.00) |
| Post-vaccine - n(%) | 6 (100) | 18 (94.7) | *0.566* | 0.94 (0.84-1.05) |
| Seroconversion- n(%) | 4 (66.7) | 11 (57.9) | *0.702* | 1.45 (0.21-9.98) |
|  |  |  |  |  |
| Flu B |  |  |  |  |
| GMT |  |  |  |  |
| Pre-vaccine (range) | 35.63 (5-320) | 44.62 (5-640) | *0.689* | -0.10(-0.37- 0.15) |
| Post-vaccine (range) | 100.79 (20-240) | 85.25 (20-1280) | *0.797* | 0.01 (-0.12-0.16) |
| GMR | 4.16 (2.71-6.43) | 3.43 (2.71-9.89) | *0.386* | 0.16 (-0.21-0.53) |
| Seroprotection- n(%) |  |  |  |  |
| Pre-vaccine - n(%) | 4 (66.7) | 13 (68.4) | *0.936* | 1.0 (0.14-7.09) |
| Post-vaccine - n(%) | 4 (66.7) | 18 (94.7) | *0.065* | 0.11 (-0.008-1.64) |
| Seroconversion- n(%) | 4 (66.7) | 5 (26.3) | *0.073* | 5.60 (0.77-40.59) |

CI, confidence interval; GMT, geometric mean titer.
