# Supplementary material for: Immunogenicity and safety of influenza vaccination in patients with juvenile idiopathic arthritis on biological therapy using the microneutralization assay
Source: Pediatr Rheumatol Online J. 2017 Aug 7;15:62. doi: 10.1186/s12969-017-0190-0 (PMC5547451; doi:10.1186/s12969-017-0190-0)
Supplement: Supplementary file 2 — Multivariable analysis for Post-vaccine seroprotection in patients with Juvenile Idiopathic Arthritis. (DOCX 18 kb) [file 12969_2017_190_MOESM2_ESM.docx]

**Additional file 2: Table S2. Multivariable analysis for Post-vaccine seroprotection in patients with Juvenile Idiopathic Arthritis**

|  | A(H1N1)pdm | | A/H3N2 | | B | |
| --- | --- | --- | --- | --- | --- | --- |
|  | **OR/ βCoeff (95% CI)** | ***p*-value** | **OR/ βCoeff (95% CI)** | ***p*-value** | **OR/ βCoeff (95% CI)** | ***p*-value** |
| JADAS | 0.005 (-0.007-0.012) | *0.42* | 0.005 (-0.007-0.01) | *0.42* | -0.02 (-0.04 - 0.002) | *0.08* |
| Leukocytes x10^9^/L | 1.12 exp-5 (0.0-0.0) | *0.46* | 6.49 exp-6 (0.0-0.0) | *0.67* | 3.55exp-5 (0.0-0.0) | *0.22* |
| Lymphocytes x10^9^/L | 0.001 (-0.005-0.006) | *0.74* | -0.002 (-0.007-0.003) | *0.47* | 0.001 (-0.009-0.012) | *0.79* |
| Neutrophils x10^9^/L | -0.001 (-0.007-0.005) | *0.78* | 0.003 (-0.003-0.009) | *0.30* | 0.008 (-0.002-0.019) | *0.12* |
| Platelets x10^9^/L | -9.6 exp -07 ( 0.0 - 0.0) | *0.001* | 0.91 (0.73 - 1.10) | *0.55* | -1.3 exp-7 (0.0 -0.0) | *0.82* |
| Hemoglobin g/L | 0.04 (-0.02-0,11) | *0.22* | 0.006 (-0.06-0.07) | *0.85* | -0.01 (-0.14-0.11) | *0.84* |
| ESR mm/h | -0.01 (-0.03- -0.005) | *0.009* | -0.001 (-0.01-0.01) | *0.91* | 0.003 (-0.02-0.03) | *0.80* |

Parameters were compared using linear or logistic regression when procedure. OR, odds ratio. CI, confidence interval. ; ESR, erythrocyte sedimentation rate
